# Retrospective chart review of inherited and idiopathic dystonia

**DOI:** 10.3389/fgene.2025.1504744

**Published:** 2025-03-11

**Authors:** A. Alakkas, H. Shinawi, J. A. Bajwa, O. Alsinaidi, A. Al-Hashim

**Affiliations:** ^1^ Movement Disorders Program, Department of Neurology, National Neuroscience Institute, King Fahad Medical City, Riyadh, Saudi Arabia; ^2^ Department of Pediatric Neurology, National Neuroscience Institute, King Fahad Medical City, Riyadh, Saudi Arabia

**Keywords:** dystonia, genetics, inherited, GCH1 variants, middle east

## Abstract

Dystonia prevalence and presentation varies both ethnically and geographically. There is a paucity of data on the clinical presentation of dystonia patients in Saudi Arabia and among Arabs. In this study we provide the largest description of dystonia patients in Saudi Arabia. In our population, majority, 42% of all patients with dystonia had an inherited dystonia, while 34.8% had idiopathic dystonia. In addition, we found 3 patients with homozygous GCH1 variants who displayed the classic phenotype of dopa-responsive dystonia. Two had Variant of Uncertain Significance that has been recently reclassified as likely pathogenic, and another novel homozygous Asp119Asn variant, not previously reported in ClinVar. It is the hope that this paper would be the first step for future prospective studies.

## 1 Introduction

Dystonia is a movement disorder characterized by sustained or intermittent muscle contractions causing abnormal postures, and/or repetitive movements (Albanese et al., 2013). Dystonia, is a highly heterogenous disorder with an ever-evolving definition and classification scheme (Albanese et al., 2013; Fahn, 2011). In 2013, an international panel of experts reviewed the definition and classification of dystonia (Albanese et al., 2013). The report classifies dystonia by a combination of two axis clinical features and etiology. Clinical features include age of onset, body distribution, disease course and associated features. Etiology is divided into inherited, acquired or idiopathic (Albanese et al., 2013; Albanese et al., 2019). The pathophysiology of dystonia is highly complex; however, genetics plays a significant role in both inherited and idiopathic dystonia (Charlesworth et al., 2013). Dystonia is a highly complex disorder with considerable genotype-phenotype variability even within members of the same family. Genetics also has a role in idiopathic dystonia where focal idiopathic dystonia tends to run in families (Charlesworth et al., 2013; Lange et al., 2021). Next-generation sequencing has resulted in the discovery of several genes causing inherited dystonia. Genetics also has a role in idiopathic dystonia where focal idiopathic dystonia tends to run in families (Charlesworth et al., 2013).

Dystonia prevalence and presentation varies both ethnically and geographically (Bailey et al., 2022). The phenotypic heterogeneity of dystonia complicates the understanding of the natural history of dystonic disorders and their response to treatment. To combat this, the dystonia collation has created a multicenter network for clinical and translational studies, unfortunately, almost all the centers included are from north American and western Europe (Kilic-Berkmen et al., 2021). To date, there is very little data on the prevalence, and clinical features of dystonia outside these regions. For example, little is known about the clinical and genetic features of patients with dystonia in Kingdom of Saudi Arabia (KSA) and the Arab world. Only one abstract described the genetic features of dystonia in Saudi Arabia (Bohlega AA et al., 2016). This paper suggested that myoclonus-dystonia might be the most common type of non-acquired dystonia in Saudi Arabia (Bohlega AA et al., 2016). Given the paucity of information on dystonic disorders in Saudi Arabia; additional studies are needed to guide clinical practice and the allocation of resources. The aim of this study is to evaluate the clinical and genetic features of patients with dystonia in a large movement disorder center. It is the hope that this paper would be the first step for future prospective studies.

## 2 Methods

### 2.1 Study design and setting

Retrospective observational study using chart review at King Fahad medical city (KFMC), Riyadh.

### 2.2 Study subjects

Patients of all ages with primary dystonia who received care at KFMC from January 2003 to February 2023.

### 2.3 Inclusion criteria

People with inherited or idiopathic dystonia (with or without a hereditary pattern) of all ages.

### 2.4 Exclusion criteria

Diagnosed with acquired dystonia.

### 2.5 Data collection procedures

Data was collected from KFMC’s epic medical record system. Following Institutional Review Board (IRB) approval, we extracted the Medical Record Numbers of patients who have dystonia documented in notes, problem list or listed as a diagnosis. All charts of patients with history of dystonia were reviewed.

The following data was collected from each chart:

1- Demographic data.
   a. date of birth, gender, nationality and city of residence.
2- Age of onset of dystonia.
   a. Infancy (birth to 2 years).
  b. Childhood (3–12 years).
  c. Adolescence (13–20 years).
  d. Adulthood (21–and older).
3- Anatomical onset of dystonia.
   a. craniocervical dystonia (Blepharospasm/Oromandibular/cervical), Larynx (Laryngeal) or Limbs (Limb dystonia).
4- Classification of dystonia.
   a. Focal (one body part) or segmental (more than 2 contiguous body parts) or multifocal (more than two non-contiguous body parts) or Hemidystonia (Ipsilateral arm and leg are involved) or generalized (more than 3 body parts).
  b. Isolated (Dystonia is the only motor feature, with the exception of tremor) or combined (Dystonia is combined with other movement disorders) or complex (Dystonia accompanied by neurologic or systemic manifestations beyond movement disorders).
  c. Inherited with proven gene origin or idiopathic.
5- Genetic disorder if identified.
6- Family history of dystonia or other psychiatric or neurological disorder.
7- Brain Magnetic resonance imaging (MRI) findings.
8- Electromyography & Nerve Conduction Studies (NCS/EMG) findings.
9- Treatment of dystonia.
   a. Current and past anti-dystonic medications.
  b. Botox injections.
  c. Surgical interventions such as Deep brain stimulation (DBS).
10- Hospitalization Data.
11- Mortality Data.

### 2.6 Ethical approval

Study was approved by KFMC IRB, protocol number 23-168.

### 2.7 Data analysis

Statistics done using SPSS software. Categorical variables are presented as frequencies and percentages, while continuous variables are presented as mean and standard deviation. To determine factors associated with the development of idiopathic and inherited dystonia in this population logistic regression and Chi-Square Tests are used. T-Test used for continues variables. A p-value of <0.05 was regarded as statistically significant.

## 3 Results

### 3.1 Study population

Demographics and clinical characteristics are summarized in Table 1. A total of 86 participants met the including and exclusion criteria. Participants are mostly male 57% with a mean age of 26.5 years (SD 19.1). They are mostly Saudi nationals 95.3% with the majority residing in the central 67% followed by southern 18.2% regions.

**TABLE 1:** Demographics and classification of dystonia.

Demographics			
		Number (Percentage)	
Age	Mean (SD)^a^	26.5 (19.1)	
Gender	Male	49 (57)	
	Female	37 (43)	
Nationality	Saudi	82 (95.3)	
	Arab Gulf States	1 (1.2)	
	Syrian	1 (1.2)	
	Indian	2 (2.3)	
Region within Saudi Arabia (For Saudi Nationals)	Central	55 (67)	
	Western	6 (7.3)	
	Eastern	3 (3.6)	
	Northern	3 (3.6)	
	Southern	15 (18.2)	
Family History	Family History of Dystonia	18 (20.9)	
	Family History of Neurological Illness	18 (20.9)	
	Consanguinity	First Cousins	25 (29.1)
		Second Cousins	3 (3.5)
		Distant Cousins	5 (5.8)
Mortality/Morbidity Data	Mortality	2 (2.3)	
	Hospitalization	None	36 (41.9)
		Less than 5	37 (43)
		5 to 10	10 (11.6)
		More than 10	2 (2.3)
		Unknown	1 (1.2)
Total Population	86		
Classification of Dystonia			
Etiology	idiopathic	39 (45.3)	
	Inherited	47 (54.7)	
Age of Onset	Infancy	21 (24.4)	
	Childhood	21 (24.4)	
	Adolescence	6 (7)	
	Adult	28 (32.6)	
	Unknown	10 (11.6)	
Body Distribution	Focal	27 (31.4)	
	Segmental	5 (5.8)	
	Multifocal	8 (9.3)	
	Hemidystonia	3 (3.5)	
	Generalized	39 (45.3)	
	Unknown	4 (4.7)	
Associated Features	Isolated	38 (44.2)	
	Combined	9 (10.5)	
	Complex	36 (41.9)	
	Paroxysmal	3 (3.5)	
Total Population	86		

^a^ Standard deviation.

20.9% had a family history of dystonia. More than a third of patients had some degree of consanguinity. Of those with consanguineous parents, 88.1% had parents who are first cousins. Consanguinity was highest in the southern region where 66.7% had consanguineous parents, but this was not statistically significantly different to other regions. Patients with inherited dystonia had a higher percentage of consanguineous parents as compared with idiopathic dystonia (63.8% vs 7.7%). In addition, having parents who are first cousins was a predictor of developing inherited dystonia as compared with idiopathic dystonia [OR 16.5 (4.3- 63.1) P < 0.001].

### 3.2 Classification of dystonia

More than half (55.8%) of patients had dystonia onset before adulthood (Table 1). Majority, 54.7% of all patients who met our inclusion and exclusion criteria had an inherited dystonia, while 45.3% had idiopathic dystonia (Table 1). Acquired dystonia made up 24.4% of original cohort, with cerebral palsy 65.4% followed by stroke 15.4% being the most common etiologies (Supplementary Table 1).

Overall, 45.3% of all patients had generalized dystonia. Body distribution was statistically significantly different between inherited and idiopathic dystonia. (P < 0.001) The Majority, 79.5% of generalized dystonia is inherited, while the majority of focal dystonia 88.9% is idiopathic in etiology. Patients with generalized dystonia were 31 times more likely to have inherited dystonia as compared with focal dystonia. (P < 0.001).

Isolated and complex dystonia had roughly equal occurrence 44.2% and 41.9% respectively (Table 1).

### 3.3 Inherited dystonia

Forty-seven individuals had inherited dystonia (Tables 1, 2). Patients with inherited dystonia were more likely to have a younger age of onset as compared with idiopathic dystonia with a mean 14.13 [(CI 10.8- 17.4) SD 11.2] and 41.4 [(CI 36.3 – 46.5), SD 15.8] respectively [Mean Difference 27.2 (CI 33 -21.4), P < 0.001] (Supplementary Figure 2). Concerning the genetic basis of inherited dystonia, 78.7% is associated with an autosomal recessive pattern of disease, whereas only 14.8% is linked to autosomal dominant inheritance, and 4.2% is attributed to X-linked recessive inheritance.

**TABLE 2:** Inherited dystonias.

Disorder			Genetic disorder	Gene	Genetic variant	Zygosity	Variant classification	Number
Primary Dystonia Genes	Isolated Dystonia		DYT-THAP1	*THAP1*	NM_018105.2:c.85C>T,p. (Arg29*) Not documented	Heterozygous	Nonsense pathogenic (class1)	(2)
	Combined Dystonia	Dopa Responsive Dystonia	DYT-GCH1	*GCH1*	NM_000161.2:c.745A>G,p.Arg249Gly NM_000161.3:c.355G>A,p.Asp119Asn Not documented	Homozygous homozygous	Likely Pathogenic (class2) VUS(class3)	(3)
			DYT-SPR	*SPR*	NM_003124.4.c.1A>G,p. (Met1?) NM_003124.4:c.354_355delinsCT,p. (Gln119*) Not documented	Homozygous Homozygous	Start-lost, likely pathogenic (class2) Nonsense pathogenic (class1)	(3)
		Dystonia Parkinsonism	Infantile-onset parkinsonism-dystonia-2	*SLC18A2*	NM_003054.6.c.1160C>T,p.Pro387Leu NM_003054.4:c.1160C>T,p. (Pro.387Leu) Not documented	Homozygous Homozygous	Missense pathogenic (class 1)	(3)
			Autosomal recessive early-onset Parkinson disease 6	*PINK1*	NM_032409.2:c.147C>T,p. (Arg492*)	Homozygous	Nonsense Pathogenic (class1)	(1)
		Myoclonus-dystonia	Myoclonus/dystonia	*SGCE*	Not documented			(1)
	Paroxysmal		ATP1A3-Related Neurologic Disorder	*ATP1A3*	Not documented NM_001256214.1:c.2444T>G,p. (Leu815Arg)	Heterozygous	Likely Pathogenic (class2)	(2)
			Paroxysmal kinesigenic dyskinesia	*PRRT2*	Not documented (2)			(2)
	Total Number (Percentage)							17 (36.1)
Heredodegenerative dystonia (Inherited Complex Dystonia)	Metabolic Disorders	Mineral metabolism /transport	Hypermagnesemia with dystonia 2	*SLC39A14*	NM_001351657.1:c.1096G>A,p. (Gly366Ser)	Homozygous	Missense VUS (class 3)	(1)
			Menkes disease	*ATP7A*	NM_000052.4:c.3141_3154del,p. (Thr1048Asnfs*12)	Hemizygous	Frameshift likely pathogenic (class2)	(1)
			NBIA type 1	*PANK2*	NM_153638.2,chr20:3869748-3899440, loss of 29.69kbpencompassing exons 1-6 of *PANK2* Seq [GRCH37]20p13 (3,835,270_3,893,281)x0 (insertional loss of 58 kb, this CNV partially encompasses *PANK2* gene Undocumented	Homozygous Homozygous	Loss, likely pathogenic (class2) Pathogenic	(3)
			NBIA type II	PLA2G6	NM_003560.2:c.1933C>T,p. (Arg645*) Not documented	homozygous	Nonsense, pathogenic (class1)	(2)
			Wilson’s Disease		Not Documented			(1)
			Woodhouse Sakati Syndrome	DCAF17	Not documented (5)			(5)
		Mitochondrial	Biotin-Thiamin Responsive Basel Ganglia disease	*SLC19A3*	NM_025243.3,c.1264A>G,p.Thr422Ala Not documented (2)	homozygous	Missense, pathogenic (class1)	(3)
			SUCLA2 mutations	SUCLA2	Not documented			(1)
		Organic acidemias	3-Methylglutaconic aciduria		Not documented			(1)
			Glutaric aciduria	GCDH	Not documented	Homozygous (3) Not documented (2)		(5)
			MethylMalonic Acidemia	*MMAA*	Not documented (2)			(2)
			L-2-hydroxyglutaric aciduria	L2HGDH	NM_024884.2:c.903T>G,p. (Tyr301*)	homozygous	Non sense, Pathogenic (class1)	(1)
		Purine metabolism	LeschNyhan syndrome	*HPRT1*	NM_000194.2:c.82_84del,p. (Tyr28del)	Hemizygous	Inframe VUS (class3)	(1)
		Lipid storage	Niemann Pick Disease type C	*NPC1*	NM_000271.5,c.2080G>C,p.Val694Leu	Homozygous	Missense,VUS (class3)	(1)
		Bilirubin metabolism	Crigler Nijar Syndrome type 1	*UGT1A1*	c.238_239insGTAC(P.Pro80ArgfsX16)	Homozygous	Outframe, ?likely pathogenic (class2)	(1)
	Ataxia Gene		CACNA1A related disorders	*CACNA1A*	NM_023035.2:c.1602G>A,p. (Met534lle)	Heterozygous	Missense VUS (class 3)	(1)
	Total Number (Percentage)							30 (63.8)
Grand Total								47

Regarding genetic etiology, majority of patients 63.8% had a heredodegenerative dystonia (inherited complex dystonia). Majority 46.7% of heredodegenerative dystonia had onset in infancy. However, most 47% of primary genetic dystonia had onset in childhood. Of the heredodegenerative dystonias, mineral metabolism disorders were most common followed by organic acidemias in our cohort. Woodhouse-Sakati Syndrome was the most common mineral metabolism disorder encountered and glutaric aciduria was the most common organic academia (Table 2). Regarding primary isolated dystonia DYT-THAP1 occurred in two individuals, we had no genetically confirmed cases of DYT-TOR1A. However, one patient had childhood onset lower limb dystonia that later generalized, this patient did not undergo genetic testing (Table 2).

### 3.4 Idiopathic dystonia

Majority of patients with idiopathic dystonia are male 61.5% but gender was not predictive of etiology (P 0.514). Idiopathic dystonias are mostly isolated 84.6%, of craniocervical anatomical onset 66.7% and focal 61.5%. Cervical dystonia is the most common form of idiopathic dystonia followed by Blepharospasm (Supplementary Table 2).

### 3.5 Investigations

46 (41.8%) of individuals had a MRI brain done, 58.3% of them had an abnormal MRI. None of the patients had a documented EMG/NCS study.

### 3.6 Treatment

94.1% of individuals are on treatment, most on benzodiazepines 45.3% followed by baclofen and anticholinergics (Supplementary Table 3). Botox was given to 37.2%, majority of which have focal dystonia 62.5%.

Bilateral Gpi DBS was performed in 5 (5.8%) individuals, current mean age is 37.6 (SD 15.3). Mean age at time of surgery is 31.2 (Range 21–59 years). All individuals who underwent DBS had generalized dystonia, 4 isolated and one combined with myoclonus. All patients had age of onset of dystonia before adulthood. The devices implanted included 4 Medtronic systems and one Abbot Medical Device.

### 3.7 Morbidly and mortality

56.9% were hospitalized at least once. Two deaths observed one due to pneumonia and another unknown. Both had NBIA type 1. The mean age of the patients who died was 8 (SD 4.2) (Supplementary Table 3).

## 4 Discussion

Dystonia prevalence and presentation varies both ethnically and geographically (Bailey et al., 2022). It is highly dependent on age of the population and gender (Defazio et al., 2004; Nutt et al., 1988). In this study we present the largest description of dystonia patients from Saudi Arabia. Our population is young (mean 26.5) and male predominant 57%. This is in keeping with the Saudi population which is slightly male predominant (50.2 percent) and has a mean age of 25 years (The General Authority for Statistics, Saudi Census, 2022).

Overall, generalized dystonia was the most common type encountered in our cohort. It is important to note that the results are unlike those of prior studies, where focal dystonia was the most common form (Meoni et al., 2020; Dressler et al., 2022; Defazio et al., 2004). For example, in the Hanover study that included 316 participants with dystonia, generalized dystonia made up only 4% of all dystonia (Dressler et al., 2022). The primary reason for the discrepancy is that our study looks at the expertise of one single center, whereas others were epidemiological studies. Thus, referral bias plays a very significant role where patients with isolated and mild dystonia might not be referred to our center. In a retrospective study done in the United Arab Emeritus they also had a predominance of focal dystonia (Waqar et al., 2024).They had equal occurrence of Males to Females and majority had dystonia onset in adulthood (Waqar et al., 2024). Their cohort only included those who were aged 12 and older. Our cohort however included individuals of all age groups and was male predominant 57%.

Regarding the classification of dystonia in our cohort, this was similar to prior studies (Albanese et al., 2013; de Carvalho Aguiar and Ozelius, 2002). We found that Idiopathic dystonia is mostly isolated, of craniocervical anatomical onset and focal. While inherited dystonia was mostly generalized and had a younger age of onset. Indicating as others have noted that genetic testing in latter group is more likely to reveal positive results.

Regarding etiology, inherited dystonia was the most common. As noted above referral bias likely plays a large role. In addition to a young population and a high degree of consanguinity. Indeed, more than a third of patients had some degree of consanguinity. Not surprisingly we found that having parents who are first cousins was a predictor of developing inherited dystonia as compared with idiopathic dystonia [OR 16.5 (4.3- 63.1) P < 0.001]. This social custom would explain that glutaric aciduria and Woodhouse Sakati syndrome, both autosomal recessive disorders were the most predominant inherited disorders. It is important to note that this is unlike a previous abstract published by our collogues at King Faisal Specialist Hospital, another tertiary care center where myoclonus dystonia was the most common inherited dystonia in their cohort (Bohlega AA et al., 2016). The total population, mean age and detailed inclusion/exclusion criteria of their data remains to be published as such one could only speculate about the discrepancy. One scenario could involve KFMC operating as both a secondary and tertiary level hospital for both adults and pediatric patients, accommodating a broader patient demographic compared to a strictly tertiary care facility.

Another distinctive aspect of our data is the predominance of autosomal recessive disorders, contrasting with the predominant autosomal dominant pattern described in the literature. In a study by Zech et al., they reported 51.9% of variants being inherited through autosomal dominant disorder and 41.6% through autosomal recessive inheritance. This difference is attributed to the high level of consanguinity within our cohort (Zech et al., 2020).

Regarding early onset inherited dystonia, in our cohort, we report three pediatric patients with homozygous GCH1 variants who displayed the classic phenotype of dopa-responsive dystonia and demonstrated a significant and sustained response to low doses of L-dopa. Among these patients, two are twin sisters, both exhibiting the homozygous Arg249Gly variant. This variant has been classified as a Variant of Uncertain Significance (VUS); however, recent classification in ClinVar suggests its likely pathogenic, particularly in a child presenting with foot dystonia. The third patient, a male, experienced symptom onset at the age of 9. Whole exome analysis revealed a novel homozygous Asp119Asn variant, not previously reported in ClinVar. Reviewing the literature, we found another patient with similar presentation to our patient, 12 years old girl experiencing dystonia, diurnal fluctuations, and consistently positive response to L-dopa treatment. She exhibited a homozygous Arg249Ser mutation, with normal levels of GCH-1 mRNA but diminished GCH-1 activity (Hwu et al., 1999).

Furukawa documented 2 cases that fell between DRD and AR GCH-1 deficient HPA. In both instances, individuals had compound heterozygous mutations in GCH-1 and exhibited significantly lower levels of BH4 and neopterin in the cerebrospinal fluid compared to those with DRD. Patient 1 exhibited both frame shift and missense mutations and he had severe phenotype, while Patient 2 had two missense mutations and his phenotype was milder. They hypothesized that the mutation in Patient 1 was more severe than that in Patient 2, leading to a more pronounced biochemical deficiency and clinical phenotype (Furukawa et al., 1998). While it was previously understood that individuals with homozygous GCH1 variants exhibit a severe neurological phenotype emerging early in life, and those with heterozygous variants present a milder phenotype known as DRD, this categorization is now less accurate, particularly due to the discovery of new variants with mild effects and the clinical finding in our patients support this notion. GCH-1 serves as a remarkable illustration demonstrating how variations in a single gene can lead to distinct phenotypes like classic dopa responsive dystonia (DRD) and atypical DRD, contingent upon the degree of mutation severity and enzyme dysfunction rather than the quantity of mutations (Lee and Jeon, 2014).

Regarding the distribution of dystonia within the various Saudi regions, it is not unexpected that the percentage of dystonia was most common in the central most populous region. However, the Southern region is second to last in terms of overall population. However, it is second in terms of the prevalence of dystonia. This could be due to higher consanguinity rates in this area, genetic makeup, and of course referral basis. Given that kinship plays a significant role, an educational campaign should emphasize the importance of premarital counseling and educate the public on the availability of preimplantation genetic testing. Indeed, prevention of this disorder will likely be cost effective as at least 58.1% had at least one hospitalization.

Regarding treatment, the majority were treated with benzodiazepines, followed by anticholinergic and baclofen. Whether the preference for benzodiazepines is due to efficacy cannot be determined by our study. The reason for the preference for benzodiazepines might be due to physician comfort and availability. Future prospective studies must be performed to determine medication efficacy in a more homogeneous population.

## 5 Conclusion

Our paper has several areas for improvement; as noted above, we cannot determine efficacy in our retrospective study. We are also unable to know the severity of the disease. In addition, we had some missing data. In addition, we cannot determine the incidence and prevalence of dystonia in Saudi Arabia. On the other hand, this is the first and largest dystonia cohort in Saudi Arabia. It is the hope that future larger national and prospective studies will be performed to better understand the predominate phenotype and response to medications.

## Data Availability

The original contributions presented in the study are included in the article/Supplementary Material, further inquiries can be directed to the corresponding author.
