# Supplementary material for: Retrospective chart review of inherited and idiopathic dystonia
Source: Front Genet. 2025 Mar 11;16:1504744. doi: 10.3389/fgene.2025.1504744 (PMC11933036; doi:10.3389/fgene.2025.1504744)
Supplement: Supplementary file 1 [file Table1.docx]

**Supplementary Tables and Figures:**

**Supplementary Figure 1:** Etiology of Acquired dystonia

**
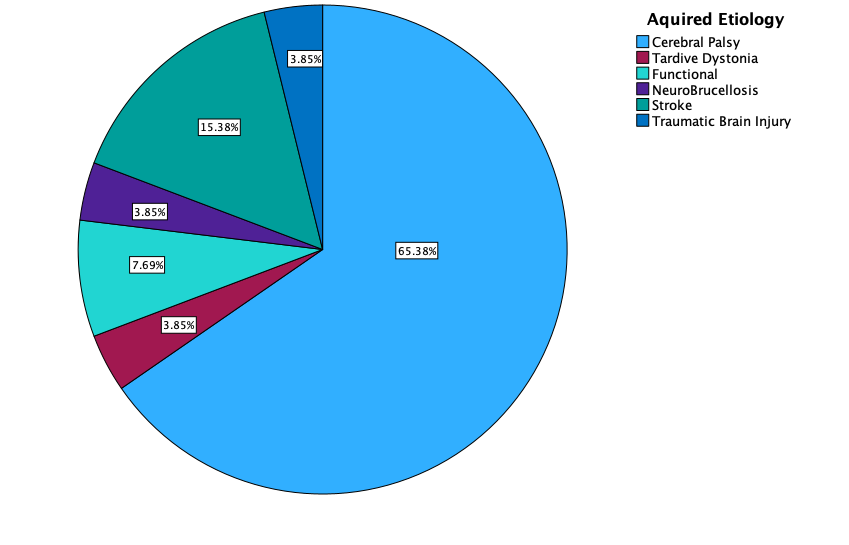
**

**Supplementary Figure 2**: Etiology of Dystonia Based on Age of Onset


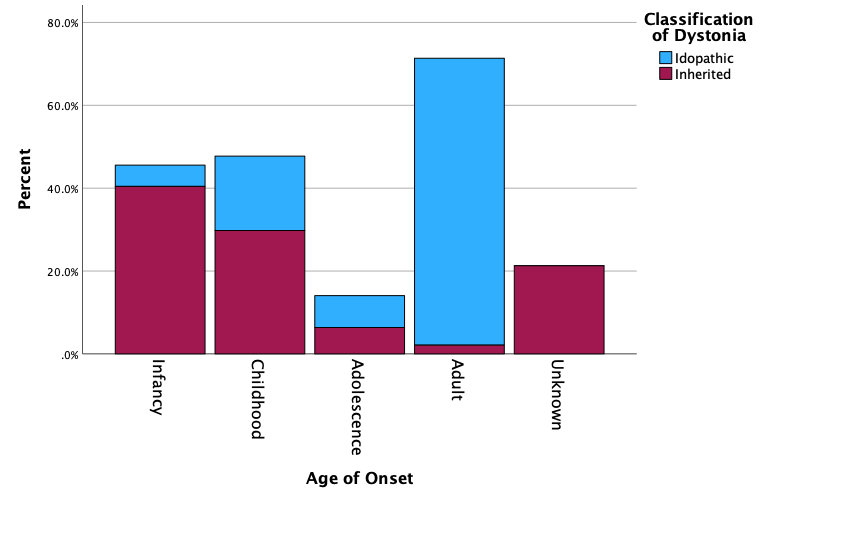


**Supplementary Table 1:** Classification of Dystonia including acquired and functional.

|  |  | Number (Percentage) |
| --- | --- | --- |
| **Etiology** | Acquired | 24 (21.4) |
|  | idiopathic | 39 (34.8) |
|  | Inherited | 47 (42) |
|  | Functional | 2 (1.8) |

**Supplementary Table 2:** Idiopathic Dystonia

|  |  | Number (Percentage) |
| --- | --- | --- |
| **Gender** | Male | 24 (61.5) |
| **Body Distribution** | Focal | 24 (61.5) |
|  | Segmental | 3 (7.6) |
|  | Multifocal | 2 (5.1) |
|  | Generalized | 8 (20.5) |
|  | Hemidystonia | 2 (5.1) |
| **Associated Features** | Isolated | 33 (84.6) |
|  | Combined | 2 (5.1) |
|  | Complex | 4 (10.3) |
| **Anatomical Onset** | Cervical | 14 (35.9) |
|  | Blepharospasm | 8 (20.5) |
|  | Oromandibular | 4 (10.3 ) |
|  | Limb Dystonia | 4 (10.3) |
|  | Unknown | 9 (23.1) |
| **Total** |  | 39 (100) |

**Supplementary table 3:** Treatment, Mortality and Hospitalization of dystonia

| **Treatment** | | | | |
| --- | --- | --- | --- | --- |
|  |  | | Number (Percentage) | |
| **Oral** | Antiepileptic | | 17 (19.8) | |
|  | Anticholinergic | | 31 (36) | |
|  | Benzodiazepines | | 39 (45.3) | |
|  | Baclofen | | 31 (36) | |
|  | Levodopa | | 17 (19.7) | |
|  | Dopamine Agonist | | 2 (2.3) | |
|  | Vesicular monoamine transporter type 2 Inhibitors | | 5 (5.8 ) | |
| **Botulinum toxin** | 32 (37.2) | | | |
| **Deep Brain Stimulation** | 5 (5.8) all Bilateral Gpi , 3 (60) Female | | | |
| **None** | 4 (4.6) | | | |
| **Mortality and Hospitalization** | | | | |
| **Mortality** | | 2 (2.3), Pneumonia 1, Unknown 1 | | |
| **Hospitalization** | | None | | 36 (41.9) |
|  |  | Less than 5 | | 37 (43) |
|  |  | 5 to 10 | | 10 (11.6) |
|  |  | More than 10 | | 2 (2.3) |
|  |  | Unknown | | 1 (1.1) |
